# The Influence of Maternal Dietary Patterns on Body Mass Index and Gestational Weight Gain in Urban Black South African Women

**DOI:** 10.3390/nu9070732

**Published:** 2017-07-11

**Authors:** Stephanie V. Wrottesley, Pedro T. Pisa, Shane A. Norris

**Affiliations:** 1 MRC/Wits Developmental Pathways for Health Research Unit, Department of Paediatrics, Faculty of Health Sciences, University of the Witwatersrand, Johannesburg, South Africa; ppisa@wrhi.ac.za (P.T.P.); shane.norris@wits.ac.za (S.A.N.); 2 Wits Reproductive Health and HIV Institute, University of the Witwatersrand, Johannesburg, South Africa

**Keywords:** dietary patterns, body mass index, gestational weight gain, urbanisation

## Abstract

Maternal pre-pregnancy body mass index (BMI) and subsequent gestational weight gain (GWG) are strong predictors of maternal and infant outcomes; however the influence of dietary patterns on BMI-specific GWG is unclear. This study identifies patterns of habitual dietary intake in urban South African women and explores their associations with first trimester BMI and GWG. Habitual dietary intake of 538 pregnant women was assessed using a quantitative food-frequency questionnaire and dietary patterns were depicted via principle component analysis. Associations between dietary patterns and BMI-specific GWG were analyzed using linear and logistic regression. Three dietary patterns were identified: Western, Traditional and Mixed. Western and Mixed diet patterns were associated with 35 g/week (*p* = 0.021) and 24 g/week (*p* = 0.041) higher GWG in normal weight and obese women respectively. Additionally, high intakes of a Traditional diet pattern were associated with a reduced odds of excessive weight gain in the total sample (OR: 0.81; *p* = 0.006) and in normal weight women (OR: 0.68; *p* = 0.003). Increased intake of a traditional diet pattern—high in whole grains, legumes, vegetables and traditional meats—and decreased intake of refined, high sugar and fat driven diets may reduce GWG (including risk of excessive weight gain) in urban South African women.

## 1. Introduction

Maternal nutritional status, principally defined by body mass index (BMI) prior to pregnancy, is a strong predictor of maternal and infant health; with both underweight (BMI < 18.5 kg/m^2^) and overweight/obesity (BMI ≥ 25 kg/m^2^) being associated with increased risk of maternal complications during pregnancy and delivery, sub-optimal fetal growth and adverse birth outcomes [1,2,3,4,5,6]. In addition to the immediate risks posed to mother and infant, adverse exposures such as malnutrition during critical periods of developmental plasticity—including fetal growth and development—have been shown to alter gene expression, thereby modifying growth and development of body tissues [7]. Such changes may lead to permanently altered metabolism and body function and increased susceptibility to non-communicable diseases (NCDs) including obesity, type 2 diabetes mellitus (T2DM) and cardiovascular disease (CVD) in later life [7,8,9,10].

In addition to pre-pregnancy BMI, weight gain during pregnancy has been shown to independently influence both maternal and infant outcomes; with inadequate weight gain, according to the Institute of Medicine (IoM) BMI-specific recommended ranges, increasing the risk of preterm birth, low birth weight and small-for-gestational age (SGA) infants [11,12]. In contrast, excessive gestational weight gain (GWG) is associated with an increased risk of pregnancy-induced hypertension, pre-eclampsia, fetal macrosomia (birth weight > 4 kg) and requiring an emergency caesarean delivery [11,13,14]. Additionally, women who gain excessive weight during pregnancy are at higher risk of postpartum weight retention, which may influence their susceptibility to developing overweight and obesity in the longer term and increase their risk profile in subsequent pregnancies [5,15,16]. 

Studies indicate that overweight and obese pregnant women are more likely to gain excessive weight than their normal-weight counterparts [17,18,19]. This is particularly relevant for low- and-middle-income countries (LMICs) such as South Africa, where the prevalence of overweight and obesity has increased substantially as a result of rapid urbanisation and a nutrition transition characterised by a shift towards typically westernised diets high in saturated fat, sugar, salt, processed foods and edible oils, as well as decreased levels of physical activity [20,21]. Pilot data from our Soweto First 1000-Day Study (S 1000) shows that urban black South African women exhibit a high clustering of disease during pregnancy, including 67% overweight and obesity, 14% gestational diabetes, 31% anaemia and 30% HIV prevalence [Norris et al., unpublished]. This high-risk profile is coupled with the poor quality, predominantly cereal-based diets that are high in energy and refined sugar and low in protein and micronutrient-rich foods such as meat, poultry, seafood, legumes and non-starchy vegetables that have been demonstrated in South African pregnant women [22].

In high-income countries (HICs) some studies have shown that a high protein: carbohydrate ratio, partly driven by a reduced added-sugar intake, is associated with lower GWG and that high adherence to a margarine, sugar and snacks dietary pattern is associated with excessive GWG when compared to low adherence to this pattern [23,24]. Additionally, in Puerto Rican women, frequent consumption of high-sugar fruit drinks was associated with excessive GWG [25]. To our knowledge, however, dietary patterns and their relationships with maternal nutritional status prior to and during pregnancy are yet to be explored in urban African populations where the associations may be further complicated by existing co-morbidities, such as the high burden of HIV, which may independently predict low pre-pregnancy BMI, poor GWG and micronutrient deficiencies [26,27,28,29].

While the relationship between maternal anthropometry prior to and during pregnancy and maternal and infant outcomes is well documented, the influence of dietary intake and patterns on BMI-specific GWG, and therefore maternal and infant outcomes, is unclear. Hence in this study, we aimed to identify patterns of habitual dietary intake in urban, black South African pregnant women and further explored their associations with first trimester BMI and GWG in the context of HIV and other socio-demographic factors.

## 2. Materials and Methods 

### 2.1. Study Setting and Participants

This study was nested within a larger longitudinal pregnancy cohort study (Soweto First 1000-Day Study; S 1000), based at the Chris Hani Baragwanath Hospital in Soweto, Johannesburg, South Africa. The overall aim of S 1000 was to understand the complexities between multiple maternal factors and fetal and infant outcomes, and to identify the levers that could optimise maternal and child health within the first 1000 days in an urban-poor African context. Women were considered eligible for inclusion in the study if they lived in Soweto, or the Greater Soweto area, and were <20 weeks gestational age at recruitment, non-epileptic, non-diabetic, 18 years or older and pregnant with singleton, naturally conceived pregnancies. Data collection for S 1000 took place at six time points during pregnancy: <14 weeks; 14–18 weeks; 19–23 weeks; 24–28 weeks; 29–33 weeks and 34–38 weeks. All the women provided written informed consent prior to their inclusion in the study and ethical approval was obtained from the University of the Witwatersrand’s Research Ethics Committee (Medical) (M120524).

### 2.2. Demographic, Health and Socio-Economic Variables

Maternal demographic and socio-economic variables were collected using an interviewer-administered questionnaire at the first visit (<14 weeks gestational age). All questionnaires were administered by trained members of research staff. Socio-economic status (SES) was assessed using a household asset index which scored each participant according to the number of assets that they possessed out of a possible 9 (electricity, radio, television, refrigerator, mobile phone, personal computer, bicycle, motorcycle/scooter, car). Maternal education was assessed according to the highest level of education (primary, secondary or tertiary) completed. Parity was defined as the number of times a subject had given birth to a fetus with a gestational age of 24 weeks or more, regardless of whether the child was born alive or was stillborn, and smoking status was assessed according to whether the participant reported smoking and/or chewing tobacco at baseline. HIV status was determined via self-report at each pregnancy visit and, if HIV-positive, the date of antiretroviral treatment (ART) initiation was recorded. According to South Africa’s national Prevention of Mother-to-Child Transmission (PMTCT) guidelines during this time, all HIV-positive pregnant women, if not already on treatment, were initiated on lifelong ART regardless of their CD4 count and therefore all HIV-positive participants were receiving ART. HIV-positive women were therefore stratified according to whether they were initiated prior to pregnancy (pre-pregnancy ART) or during the current pregnancy (antenatal ART). 

### 2.3. Anthropometry

Maternal weight was measured to the nearest 0.1 kg using a digital scale at each pregnancy visit and maternal height was measured to the nearest 1 mm using a wall-mounted Stadiometer (Holtain, UK) at the first visit. Weight at the first visit (<14 weeks) was used as a proxy for pre-pregnancy weight and, together with height, used to calculate maternal BMI (weight (kg)/height (m^2^)). As there were no underweight women in this sample, BMI was classified as either normal weight (18.5–24.9 kg/m^2^), overweight (25–29.9 kg/m^2^) or obese (≥30.0 kg/m^2^). GWG (kg/week) was calculated as: (weight at last visit − weight at recruitment)/duration of follow-up. Women were classified as having inadequate, adequate or excessive GWG using the BMI-specific IoM recommended ranges [30].

### 2.4. Dietary Intake

Habitual dietary intake was assessed using an interviewer-administered quantitative food-frequency questionnaire (QFFQ) during the second pregnancy visit (14–18 weeks). The QFFQ has been extensively utilised in this setting and results are published elsewhere [31,32]. Data was collected retrospectively on the previous week’s intake of local South African foods, including convenience food products. Frequency and quantity—defined using a combination of household measures, two-dimensional life-size drawings of foods and utensils and three-dimensional food models, as described by Steyn et al. [33], and then converted to an average intake in grams per day—of each food item consumed was recorded during the interview and captured electronically using REDCap electronic data capture tools hosted at The University of the Witwatersrand [34].

### 2.5. Statistical Analysis

Data were analysed for 538 pregnant women with complete data using STATA 13.0 (StataCorp, College Station, TX, USA). Principal component analysis (PCA) was the dimension reduction technique used to depict dietary patterns. This is a statistical method that groups variables—in this case food items/groups—based on the level of correlation with each other to form new linear components that reflect the combinations (patterns) of intake in a population [35,36]. PCA was conducted on the weekly frequency of consumption of the food items listed in the QFFQ, classified into 49 food groups according to their nutrient composition and usage. These groupings were based on those used and described extensively by Crozier et al. [37,38]. PCA was applied using orthogonal (varimax) rotation and the Kaiser-Meyer-Olkin measure of sampling adequacy (0.65) and Bartlett’s test of sphericity (*p* < 0.001) confirmed PCA to be an appropriate dimension reduction technique to apply in this sample. Eigen values, as well as their visual inflections on a scree plot, and the total variance explained (percentages) were used to identify retained patterns. As described elsewhere, foods or food groups that had factor loading scores ≥ 0.2 on the PCA matrix reflected strong associations with principal components and these were used to name dietary patterns [24]. A dietary pattern score for each pattern was generated for all participants by multiplying factor loadings by the standardised intake of each food/food group and then summing these. Mean factor scores for the patterns were zero; with positive and negative scores therefore representing high and low intakes of each dietary pattern respectively [36].

Maternal characteristics of the sample are presented as median (interquartile range) and percentages (%) for continuous and categorical variables respectively. The Kruskal-Wallis and Chi^2^ tests were used to compare all continuous and categorical variables respectively across the three IoM defined BMI-specific GWG categories; namely inadequate, adequate and excessive weight gain. Potential covariates for the associations between dietary patterns and GWG were identified by comparing factor scores for each pattern using either a t-independent test (two categories) or analysis of variance (ANOVA) (more than two categories) according to the following maternal factors: maternal age, parity, HIV status, education, marital status, SES, BMI at recruitment and GWG. Furthermore, in cases where there were more than two categories for a given characteristic, a Tukey post-hoc test was carried out to identify where the differences occurred between categories (data presented as a supplementary Table S1).

The associations between depicted dietary pattern scores and GWG were tested using multivariable linear regressions and multinomial logistic regressions for the total sample and then across three BMI categories; namely normal weight, overweight and obese. Linear regression analyses were based on continuous dietary pattern scores (exposure) and GWG (g/week) (outcome) and logistic regression analyses were based on dietary pattern scores (exposure) and adequacy of GWG according to the IoM recommended ranges (inadequate and excessive GWG vs. adequate GWG; reference) (outcome). Due to the fact that the diet patterns are not independent and it is possible for individuals to have high scores for multiple patterns, all regression analyses (crude and adjusted models) included all diet patterns. 

Regression coefficients for linear regressions are therefore presented for a 1 SD increase in dietary pattern scores across three models namely; Model 1 (M1): crude analysis adjusted for the other diet patterns; Model 2 (M2): M1 adjusted for covariates shown to be significantly associated with dietary patterns and Model 3 (M3): M2 adjusted for total energy intake. Due to the similarities observed between M1 and M2 during logistic regression analyses, odds ratios are presented for only two models; i.e., replacing M2 with M3.

## 3. Results

### 3.1. Maternal Characteristics

Maternal demographic, health, SES and anthropometric variables stratified according to IoM BMI-specific GWG categories are presented in Table 1. 24%, 21% and 55% of women were categorised as experiencing inadequate, adequate and excessive GWG respectively. Median maternal age decreased across GWG categories (*p* = 0.038); however there were no significant differences in the distribution of women across age range categories between the GWG groups. HIV status was significantly associated with GWG, with HIV-negative women being more likely to exhibit excessive weight gain and HIV-positive women more likely to exhibit inadequate weight gain; regardless of whether they had been initiated on ART prior to the current pregnancy. Household SES increased across categories of GWG. Median BMI was lowest in women who gained adequate weight during pregnancy, with the prevalence of excessive weight gain being significantly higher in overweight and obese women than in their normal weight counterparts who were more likely to gain either inadequate or adequate weight. There were no significant differences in parity, smoking status, education or marital status across GWG adequacy groups.

### 3.2. Identification and Description of Depicted Dietary Patterns

Three distinct dietary patterns were identified which explained 20.5% of the variation in food intakes (Table 2 and Supplementary Figure S1). The first principal component (PC) was characterised by high factor loadings for energy dense, processed, high sugar/fat foods (white bread, processed and red meat, roast potatoes and chips, sweets and chocolate, soft drinks and cheese) and was labelled the “Western” pattern (8.7% variance). The second PC, labelled the “Traditional” pattern, had high factor loadings for beans and legumes, vegetables, traditional meats and porridge/pap (6.4% variance). The final PC was heterogeneous in composition, with both classically healthy foods such as whole grains, nuts and dairy as well as high sugar items (added sugar and sweet spreads) loading high (5.4% variance). This was labelled the “Mixed” pattern. 

### 3.3. Associations between Maternal Demographic, Health, SES and Anthropometric Characteristics and Dietary Pattern Scores

Of the maternal demographic, HIV status, SES and anthropometric variables tested as potential confounders or effect modifiers of the association between dietary patterns and GWG, only parity and marital status were shown to conclusively influence diet; with younger women with no previous births consuming a more Western pattern and married/cohabiting women consuming a more Traditional diet vs. single women who scored higher for the Western diet pattern (data not shown; Supplementary Table S1). 

### 3.4. The Effect of Identified Dietary Patterns on Gestational Weight Gain

In the total sample of pregnant women, only the Mixed diet pattern showed a significant, positive association with rate of GWG in both crude (M1: 22 (7.3) g/week; *p* = 0.003) and adjusted models (M2: 22 (7.3) g/week; *p* = 0.003; M3: 22 (7.6) g/week; *p* = 004) (Table 3). This positive association was maintained in obese women for all models (M1: 25 (11.4) g/week; *p* = 0.029; M2: 23 (11.4) g/week; *p* = 0.042; M3: 24 (11.6) g/week; *p* = 0.041), but was not shown in normal weight or overweight women. While the Western diet pattern was not associated with rate of GWG in the total sample of pregnant women or in the overweight/obese subsamples, it was significantly associated with higher weight gain in normal weight women in all models. The Traditional pattern showed significant, inverse associations with GWG in M1 (−27 (11.1) g/week; *p* = 0.015) and in M2 (−27 (11.5) g/week; *p* = 0.02); however, this association was no longer significant after adjustment for total energy intake. 

During crude logistic regression analyses, a higher Western diet pattern score was associated with an increased odds of excessive weight gain in normal weight women (M1: 1.30; *p* = 0.014) (Table 4). However, this association was not maintained after adjustment for covariates. A 1SD increase in Traditional diet pattern score was associated with a 19% reduction in the odds of excessive weight gain in the total sample after adjustment for covariates (M2: 0.81; *p* = 0.006). This association was similarly shown in the normal weight women in both crude (M1: 0.77; *p* = 0.021) and adjusted models (M2: 0.68; *p* = 0.003). Mixed diet pattern scores were not associated with GWG adequacy in the total sample, or across any BMI categories.

## 4. Discussion

To our knowledge this is the first study to identify dietary patterns in urban, black South African pregnant women and to explore their relationships with first trimester BMI and GWG. We found that, higher intake of a Mixed diet pattern was associated with increased rate of GWG in both the total sample and in the subgroup of obese women, while a Western diet pattern was associated with higher weight gain in normal weight women. In addition, high intake of a Traditional diet pattern was associated with a reduction in the odds of excessive GWG, predominantly in normal weight women.

Observed positive associations between both the Western and Mixed diet patterns and rate of GWG are supported by Uusatilo et al. who found that greater adherence to a Fast Food diet pattern—characterised by high intakes of fast food items such as hamburgers and pizza, as well as sweets, soft drinks and added sugar—was positively associated with maternal weight gain [39]. Although our Mixed diet pattern was heterogeneous in nature, the high added sugar content may be driving this association and potentially negate some of the benefits of the “healthier” items such as whole grains and nuts. This is supported by previous studies in which higher intakes of a Margarine, Sugar and Snacks diet pattern and more frequent consumption of high-sugar fruit drinks have been positively associated with excessive GWG [24,25].

The association identified between the Traditional diet pattern and adequacy of GWG is unique to this study and, to our knowledge, has not been shown in other settings. Previous studies have reported contrasting findings for associations between the more “healthy” dietary patterns identified; with Uusitalo et al. showing no association between a Healthy diet pattern (high in vegetables, fish, poultry, legumes, fruit and eggs) and rate of GWG and Tielemans et al. showing a positive association between a Vegetable, Oil and Fish pattern and GWG [24,39]. However, when assessing diet using a quality index (i.e., level of adherence to a pre-defined “healthy” diet; in this case a diet high in fruit, vegetables, whole grains, potatoes, fish, game and milk) Hillesund et al. found that diets of higher nutrient density and healthier macronutrient distribution were associated with reduced odds of excessive GWG in normal weight women [40]. As with our study, the associations between maternal diet and GWG adequacy were not seen in overweight/obese women.

While high Mixed diet pattern scores were significantly associated with higher rate of weight gain in both the total sample and the obese subgroup, we found that high Western pattern intake was only associated with higher GWG in the normal weight women. In addition, the reduction in odds of excessive GWG associated with a Traditional diet pattern in the total sample was evident in normal weight, but not in overweight or obese women after stratification. Differential associations between diet patterns and GWG across BMI categories have been shown previously, with both Tielemans et al. and Hillesund et al. finding that the associations between diet patterns and either rate of weight gain or GWG adequacy were specific to normal weight women [24,40]. Due to the complexity of the relationship between diet patterns, BMI and GWG, the reason for this is difficult to ascertain; however it is possible that the effects of diet quality on GWG may be somewhat negated if quantity remains high; particularly in overweight/obese women who are already at an increased risk of higher weight gain. In addition, overweight and obese women have a reduced capacity for weight gain according to IoM recommended ranges than their normal weight counterparts and this may limit the effect of improved diet quality on adequacy of weight gain in these women [40]. Differences may also be a result of the higher degree of under-reporting of dietary intake associated with increasing BMI [41]. Although under- and over-reporting has largely focused on overall energy and macronutrient intakes, women have been shown to preferentially under-report foods that they believe to be unhealthy or fattening—such as high sugar and snack foods—which may result in greater inaccuracies in the dietary data reported by overweight and obese women when compared to those of normal weight [42,43].

As previously mentioned, the relationship between diet and weight gain during pregnancy is complex; as reflected by the effect of adjusting for identified covariates in the regression models. Both dietary patterns and GWG are associated with a number of other sociodemographic and health factors which may confound or modify the associations between identified patterns and weight gain. In our sample of pregnant women we show that, although HIV status is not associated with dietary patterns, HIV-positive women are more likely to gain inadequate weight during pregnancy than their HIV-negative counterparts; regardless of whether they were initiated on ART prior to, or during, the current pregnancy. Although inadequate weight gain is not the key focus in this highly overweight/obese population, the high prevalence of HIV (34%) and increased risk of adverse birth outcomes associated with inadequate weight gain during pregnancy [1] highlight a need to tailor dietary advice to promote higher weight gain where necessary, while potentially monitoring weight gain throughout pregnancy, in this vulnerable population.

Alongside HIV status, our results highlight additional factors which should be considered when tailoring advice or interventions to promote improved diet quality and optimal weight gain in urban black South African pregnant women. We found that younger, nulliparous, single women had higher adherence to a Western (“less healthy”) pattern, while women who had a previous birth and were married or cohabiting had higher adherence to a Traditional (“healthier”) diet pattern. Similar results have been shown previously for age and marital status [36,44,45].

Although less convincing than the contrasting associations we have shown between Western and Traditional diet patterns and both parity and marital status, we found that Western diet pattern scores were higher in more educated women. This is opposite to relationships shown between patterns of dietary intake and education in HICs; with more educated women being more likely to consume the studies respective “healthy” diet pattern in these cases [36,44]. This may be due to the influence of urbanisation in our setting, where improved education and higher household SES have been positively associated with obesity risk; potentially as a result of increased intakes of more refined, high fat foods and reduced levels of physical activity [46,47]. This is reflected by the positive association identified between household SES and the Mixed diet pattern in our study which was high in full fat dairy, margarine and added sugar.

While dietary intake has been explored in pregnant South African women, studies have been limited to the use of traditional analyses that assess macro- and micronutrient or food item intakes in isolation rather than overall patterns of dietary intake. This is a widely recognised flaw in the classic approaches to dietary assessment, as diets consist of multiple combinations of foods/nutrients consumed together, nutrient intakes are highly correlated and individual nutrients may have very small effects [35,36]. This makes independent nutrient-outcome associations difficult to ascertain, while limiting their practicality and relevance for use in public-health messaging. Use of PCA to depict dietary patterns therefore provides strength to our study in that we are able to capture both the diversity and quality of population diets and therefore contribute to a more comprehensive understanding of the relationships between habitual eating behaviours and outcomes of interest. In addition, it provides a more accessible basis for comparison with food based dietary guidelines (FBDGs), as well as for recommendations to the public who may have limited understanding of the nutrient composition of foods [35,45]. However, this approach is not free from limitations; with the majority being linked to the subjective nature of decisions made on the grouping of food items and formatting of the input variables, as well as the number and naming of the derived patterns [48,49].

Further limitations of our study included the use of BMI at recruitment to classify maternal pre-pregnancy BMI status. Although first trimester weight has been identified as an adequate proxy for pre-pregnancy weight, BMI specific differences in the amount of weight gained—although low overall—have been shown in black women and may result in a degree of misclassification [50,51]. In addition, differences in the timing of anthropometric measurements resulted in variations in follow-up duration for GWG assessment between participants which were dependent on gestational age at both recruitment and the final visit prior to delivery. However, this was addressed through using the rate of gain per week rather than total gain through pregnancy; thereby increasing accuracy and comparability of GWG in the sample. While we included a number of covariates in our study which were shown to be associated with diet pattern(s) and/or GWG, we did not have data on all behavioural factors which may influence these relationships; for example physical activity which, alongside change in dietary intake and smoking, has been proposed as a determinant of GWG [52]. Lastly, although our sample size was sufficiently robust for analysis of associations between diet and GWG in the total sample, the numbers of women within specific BMI categories were substantially lower and may be a contributing factor to the differences in the associations observed after stratification.

## 5. Conclusions

Our findings suggest that increased intakes of a Traditional diet pattern—high in whole grains, legumes, vegetables and traditional meats and low in processed foods—and reduced intakes of highly refined dietary patterns—characterized by high sugar, fat and convenience food products—may significantly reduce GWG (including odds of excessive weight gain in urban black South African women. However, due to the high prevalence of overweight and obesity in this population, these results emphasize the need for early intervention to improve diet quality and promote a healthy weight prior to conception. This would not only reduce the risk of excessive GWG inherently associated maternal obesity, but enhance the effects of good quality diets on weight gain adequacy during pregnancy to potentially improve both maternal and infant outcomes.

## Figures and Tables

**Table 1:** Maternal characteristics of South African women according to Institute of Medicine (IoM) BMI-specific gestational weight gain categories.

Variable	BMI-Specific Gestational Weight Gain (kg/Week)				*p*-Value ^a^
	Total	Inadequate	Adequate	Excessive	
	(*n* = 538)	(*n* = 128)	(*n* = 113)	(*n* = 297)	
***Maternal characteristics***					
Age, year	30 (25–34)	31 (27–36)	30 (26–35)	29 (25–34)	**0.038**
<25	115 (21)	19 (15)	25 (22)	71 (24)	0.216
25–29	153 (29)	39 (30)	28 (25)	86 (29)	
30–34	137 (26)	31 (24)	31 (28)	75 (25)	
35–39	99 (18)	25 (20)	23 (20)	51 (17)	
≥40	34 (6)	14 (11)	6 (5)	14 (5)	
Parity					
Para 0	134 (25)	22 (17)	33 (29)	79 (26)	0.189
Para 1	236 (44)	62 (49)	44 (39)	130 (44)	
Para ≥2	168 (31)	44 (34)	36 (32)	88 (30)	
HIV status					
HIV-negative	357 (66)	66 (52)	72 (64)	219 (74)	**<0.001**
HIV-positive (pre-pregnancy ART)	65 (12)	22 (17)	15 (13)	28 (9)	
HIV-positive (antenatal ART)	116 (22)	40 (31)	26 (23)	50 (17)	
Smokes/chews tobacco					
No	467 (87)	108 (84)	100 (88)	259 (87)	0.612
Yes	71 (13)	20 (16)	13 (12)	38 (13)	
***Socioeconomic characteristics***					
Maternal education					
Primary	10 (2)	4 (3)	2 (2)	4 (1)	0.214
Secondary	377 (70)	96 (75)	82 (72)	199 (67)	
Tertiary	151 (28)	28 (22)	29 (26)	94 (32)	
Marital status ( *n* = 529)					
Single	326 (62)	73 (59)	70 (62.5)	183 (62)	0.771
Married/cohabiting	203 (38)	51 (41)	42 (37.5)	110 (38)	
Household SES					
Low	77 (14)	25 (20)	16 (14)	36 (12)	**0.048**
Medium	430 (80)	100 (78)	86 (76)	244 (82)	
High	31 (6)	3 (2)	11 (10)	17 (6)	
***Anthropometry***					
Height, cm	158.3 (154.5–162.4)	156.7 (153.5–161.3)	158.8 (154.8–162.8)	159 (154.8–163)	**0.042**
Weight at recruitment, kg (<14 weeks)	68.8 (59.7–78.4)	66.9 (57.7–76.4)	64 (56.5–76.4)	70.8 (62–79.3)	**<0.001**
BMI at recruitment, kg/m^2^ (<14 weeks)	27.5 (23.9–31.0)	27.3 (23.28–30.9)	25.4 (22.2–29.5)	28.2 (25.1–31.2)	**0.001**
Normal weight (BMI 18.5–24.9 kg/m^2^)	182 (34)	54 (42)	54 (48)	74 (25)	**<0.001**
Overweight (BMI 25–29.9 kg/m^2)^	190 (35)	38 (30)	32 (28)	120 (40)	
Obese (BMI ≥30 kg/m^2)^	166 (31)	36 (28)	27 (24)	103 (35)	
GWG, kg/week	0.40 (0.27–0.55)	0.14 (0.06–0.22)	0.32 (0.27–0.42)	0.54 (0.42–0.64)	**<0.001**

Data are summarised as median (IQR) or *n* (%); IoM gestational weight gain ranges (kg/m^2^), Inadequate: normal weight < 0.35, overweight < 0.23, obese < 0.17; Adequate: normal weight 0.35–0.50, overweight 0.23–0.33, obese 0.17–0.27; Excessive: normal weight > 0.50, overweight > 0.33, obese > 0.27; ^a^ Kruskal-Wallis test (continuous variables), Chi^2^ test (categorical variables).

**Table 2:** Factor loadings of various foods or food groups characteristic to the principal dietary components identified in pregnant South African women (*n* = 538).

Food or Food Group	PC1	PC2	PC3
	*Western Pattern*	*Traditional Pattern*	*Mixed Pattern*
White bread	0.318		
Cheese and cottage cheese	0.244		
Red meat	0.212		
Processed meat	0.335		
Roast potatoes and chips	0.353		
Sweets and chocolate	0.245		
Soft drinks	0.249		
Miscellaneous (soup powder, condiments, sauces, etc.)	0.325		
Maize, sorghum and oat porridge		0.229	
Offal and traditional meats		0.251	
Salad vegetables		0.262	
Green vegetables		0.336	
Root vegetables		0.248	
Other vegetables		0.340	
Vegetable dishes		0.251	
Beans and pulses		0.273	
Boiled and baked potatoes		0.207	
Other fruit		0.211	
Brown and wholemeal bread			0.325
Breakfast cereals			0.208
Full-fat milk			0.368
Reduced-fat spread			0.307
Nuts and nut spreads			0.259
Added sugar (teaspoons)			0.361
Sweet spreads			0.327
Decaffeinated tea and coffee			0.211
Explained variance (%)	8.7	6.4	5.4
Cumulative explained variance (%)	8.7	15.1	20.5

Foods or food groups presented had factor loadings ≥ 0.2 and were therefore used to describe each dietary pattern.

**Table 3:** Associations between dietary pattern scores and rate of gestational weight gain in South African women.

Dietary Pattern	Gestational Weight Gain (g/Week)											
	*Total (n = 538)*			*Normal Weight (n = 182)*			*Overweight (n = 190)*			*Obese (n = 166)*		
	β	SE	*p*-Value ^a^	β	SE	*p*-Value ^a^	β	SE	*p*-Value ^a^	β	SE	*p*-Value ^a^
***Western***												
Model 1	8	5.8	0.181	27	10.6	**0.013**	5	9.9	0.588	−7	9.3	0.469
Model 2	4	5.9	0.527	25	10.9	**0.025**	−1	10.2	0.887	−7	9.4	0.443
Model 3	4	8.6	0.640	35	14.9	**0.021**	−8	14.5	0.593	−4	15.9	0.795
***Traditional***												
Model 1	−12	6.8	0.077	−27	11.1	**0.015**	−3	12.0	0.787	−5	11.8	0.683
Model 2	−7	7.0	0.311	−27	11.5	**0.020**	4	12.5	0.751	−1	12.0	0.935
Model 3	−7	7.5	0.353	−23	12.2	0.063	2	13.1	0.900	1	13.7	0.964
***Mixed***												
Model 1	22	7.3	**0.003**	17	13.3	0.215	20	12.8	0.111	25	11.4	**0.029**
Model 2	22	7.3	**0.003**	15	13.7	0.281	21	12.9	0.107	23	11.4	**0.042**
Model 3	22	7.6	**0.004**	19	14.4	0.182	18	13.7	0.187	24	11.6	**0.041**

Values are regression coefficients (β) with standard errors (SE) that represent the difference in rate of gestational weight gain (g/week) for a 1SD increase in dietary pattern scores; ^a^ Multivariable linear regression analyses; significant results presented in bold (*p* < 0.05); Model 1: crude analysis, adjusted for other diet patterns; Model 2: M1 adjusted for parity and marital status; Model 3: M2 adjusted for total energy intake.

**Table 4:** Associations between dietary pattern scores and adequacy of gestational weight gain in South African women.

Dietary Pattern	Gestational Weight Gain Category															
	*Total (n = 538)*				*Normal Weight (n = 182)*				*Overweight (n = 190)*				*Obese (n = 166)*			
	Inadequate		Excessive		Inadequate		Excessive		Inadequate		Excessive		Inadequate		Excessive	
	OR	95% CI	OR	95% CI	OR	95% CI	OR	95% CI	OR	95% CI	OR	95% CI	OR	95% CI	OR	95% CI
***Western***																
Model 1	0.98	0.85; 1.12	1.06	0.94; 1.19	0.98	0.78; 1.24	1.30	1.06; 1.61	0.94	0.72; 1.23	1.05	0.84; 1.31	0.98	0.77; 1.25	0.91	0.74; 1.13
*p*-value ^a^		0.717		0.334		0.877		**0.014**		0.658		0.666		0.892		0.403
Model 2	0.82	0.66; 1.02	0.84	0.70; 1.01	0.81	0.58; 1.12	1.07	0.78; 1.45	0.86	0.56; 1.31	0.80	0.56; 1.15	0.83	0.52; 1.30	0.70	0.47; 1.05
*p*-value ^a^		0.077		0.068		0.202		0.682		0.471		0.224		0.410		0.084
***Traditional***																
Model 1	1.03	0.89; 1.20	0.90	0.79; 1.03	0.97	0.79; 1.21	0.77	0.62; 0.96	1.09	0.81; 1.48	0.90	0.69; 1.17	1.16	0.83; 1.61	1.18	0.89; 1.57
*p*-value ^a^		0.700		0.132		0.803		**0.021**		0.560		0.426		0.383		0.256
Model 2	0.90	0.75; 1.07	0.81	0.69; 0.94	0.85	0.67; 1.09	0.68	0.53; 0.87	0.92	0.64; 1.33	0.77	0.56; 1.04	1.04	0.70; 1.54	1.07	0.77; 1.50
*p*-value ^a^		0.216		**0.006**		0.205		**0.003**		0.675		0.090		0.853		0.681
***Mixed***																
Model 1	0.95	0.80; 1.13	1.03	0.90; 1.19	0.94	0.72; 1.21	0.87	0.68; 1.12	1.00	0.70; 1.44	1.31	0.97; 1.78	0.95	0.69; 1.30	0.99	0.69; 1.30
*p*-value ^a^		0.557		0.651		0.619		0.273		0.994		0.077		0.744		0.952
Model 2	0.89	0.74; 1.07	0.96	0.82; 1.12	0.83	0.61; 1.12	0.77	0.58; 1.03	0.96	0.64; 1.44	1.25	0.89; 1.75	0.93	0.67; 1.28	0.94	0.71; 1.25
*p*-value ^a^		0.231		0.597		0.213		0.078		0.836		0.201		0.648		0.669

Values are relative risk ratios with 95% confidence intervals that represent the association between a 1 SD increase in dietary pattern scores and inadequate or excessive gestational weight gain relative to adequate weight gain (reference); ^a^ Multinomial logistic regression analyses; significant results are presented in bold (*p* < 0.05); Model 1: crude analysis, adjusted for other diet patterns; Model 2: M1 adjusted for parity, marital status and total energy intake.

## Supplementary Materials

The following are available online at www.mdpi.com/2072-6643/9/7/732/s1, Figure S1: Scree plot of eigenvalues after principal component analysis; Table S1: Associations between maternal characteristics and dietary pattern scores (*n* = 538).
