# Supplementary material for: The Influence of Maternal Dietary Patterns on Body Mass Index and Gestational Weight Gain in Urban Black South African Women
Source: Nutrients. 2017 Jul 11;9(7):732. doi: 10.3390/nu9070732 (PMC5537846; doi:10.3390/nu9070732)
Supplement: Supplementary file 1 [file nutrients-09-00732-s001.zip › nutrients-197823-supplementary.pdf]

Supplementary figure S1: Scree plot of eigenvalues after principal component analysis

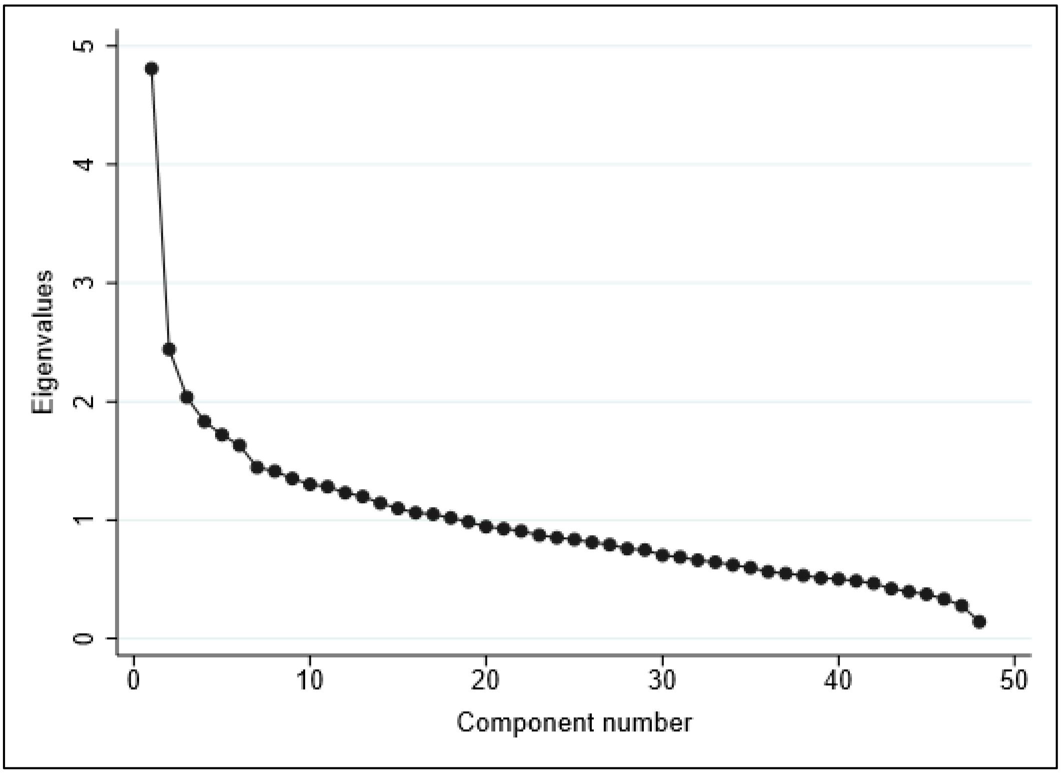

Supplementary table S1: Associations between maternal characteristics and dietary pattern scores (n=538)

Variable	Dietary pattern		
	Western	Traditional	Mixed
Maternal characteristics			
Age, y			
<25	0.68 ± 2.27	-0.16 ± 1.78	0.10 ± 1.66
25-29	0.21 ± 2.12	-0.21 ± 1.81	-0.06 ± 1.52
30-34	-0.22 ± 1.96[b]	0.10 ± 1.68	0.06 ± 1.69
35-39	-0.47 ± 1.64[b]	0.37 ± 1.84	-0.00 ± 1.65
≥40	-0.97 ± 1.36[bc]	0.03 ± 1.36	-0.30 ± 1.41
P-value[a]	**<0.001**	0.094	0.745
Parity			
Para 0	0.15 ± 1.97	-0.39 ± 1.40	-0.11 ± 1.38
Para 1	0.23 ± 2.12	0.22 ± 1.91[e]	0.20 ± 1.74
Para ≥2	-0.44 ± 1.93[d]	-0.00 ± 1.74	-0.19 ± 1.57[f]
P-value[a]	**0.003**	**0.006**	**0.034**
HIV status			
HIV-negative	-0.06 ± 2.05	-0.03 ± 1.67	0.05 ± 1.56
HIV-positive (pre-pregnancy ART)	0.23 ± 2.19	0.40 ± 2.20	-0.02 ± 1.81
HIV-positive (antenatal ART)	0.05 ± 1.92	-0.13 ± 1.73	-0.14 ± 1.64
P-value[a]	0.560	0.126	0.538
Smokes/chews tobacco			
No	-0.02 ± 2.06	0.03 ± 1.76	0.06 ± 1.63
Yes	0.15 ± 1.91	-0.23 ± 1.71	-0.42 ± 1.40
P-value[a]	0.512	0.239	**0.019**
Socioeconomic characteristics			
Maternal education			
Primary	-1.54 ± 1.29	-0.41 ± 1.29	0.15 ± 1.92
Secondary	-0.07 ± 2.05	-0.01 ± 1.74	-0.04 ± 1.64
Tertiary	0.28 ± 2.01[g]	0.06 ± 1.84	0.08 ± 1.53
P-value[a]	**0.011**	0.704	0.700
Marital status (n=509)			
Single	0.15 ± 2.15	-0.14 ± 1.66	0.07 ± 1.61
Married/cohabiting	-0.24 ± 1.84	0.20 ± 1.88	-0.08 ± 1.61
P-value[a]	**0.035**	**0.032**	0.315
Household SES			
Low	-0.10 ± 2.15	0.00 ± 1.75	-0.67 ± 1.59
Medium	0.00 ± 2.01	0.03 ± 1.77	0.11 ± 1.61[h]
High	0.22 ± 1.82	-0.44 ± 1.57	0.15 ± 1.30[i]
P-value[a]	0.759	0.353	**<0.001**
Anthropometry			
BMI at recruitment, kg/m² (<14 weeks)			
Normal weight	0.06 ± 2.06	0.10 ± 1.91	0.09 ± 1.62
Overweight	-0.09 ± 1.98	-0.12 ± 1.63	-0.15 ± 1.47
Obese	0.03 ± 2.09	0.02 ± 1.72	0.06 ± 1.75
P-value[a]	0.736	0.460	0.289

Gestational weight gain (GWG), kg/w			
Normal weight			
Inadequate (<0.35)	-0.29 ± 2.02	0.22 ± 2.11	0.04 ± 1.73
Adequate (0.35-0.50)	-0.09 ± 2.01	0.43 ± 2.02	0.27 ± 1.51
Excessive (>0.50)	0.44 ± 2.09	-0.21 ± 1.63	0.01 ± 1.63
P-value[a]	0.117	0.152	0.313
Overweight			
Inadequate (<0.23)	-0.31 ± 1.55	0.10 ± 1.50	-0.47 ± 1.40
Adequate (0.23-0.33)	-0.19 ± 1.93	-0.08 ± 1.66	-0.48 ± 1.20
Excessive (>0.33)	0.01 ± 2.12	-0.20 ± 1.67	0.04 ± 1.52
P-value[a]	0.656	0.597	0.064
Obese			
Inadequate GWG, kg/w (<0.17)	0.21 ± 2.32	0.06 ± 2.05	-0.01 ± 1.96
Adequate GWG, kg/w (0.17-0.27)	0.18 ± 2.29	-0.28 ± 1.37	0.03 ± 1.50
Excessive GWG, kg/w (>0.27)	-0.06 ± 1.97	0.09 ± 1.69	0.10 ± 1.75
P-value[a]	0.742	0.614	0.951

Dietary pattern scores presented as mean ±SD

Normal weight, BMI 18.5-24.9kg/m^2; overweight, BMI 25-29.9kg/m^2; obese, BMI ≥30kg/m^2

[a] T-independent test (two groups) or analysis of variance (ANOVA) (more than two groups)

[b] Tukey post-hoc test: P<0.01 vs. <25 years [c] Tukey post-hoc test: P<0.05 vs. 25-29 years

[d]Tukey post-hoc test: P<0.05 vs. para 0; P<0.01 vs. para 1 [e]Tukey post-hoc test: P<0.01 vs. para 0 [f]Tukey post-hoc test: P<0.05 vs. para 1

[g]Tukey post-hoc test: P<0.05 vs. primary education

[h]Tukey post-hoc test: P<0.001 vs. low SES [i]Tukey post-hoc test: P<0.05 vs. low SE
